# Nomogram based on clinical and brain computed tomography characteristics for predicting more than 5 cerebral microbleeds in the hypertensive population

**DOI:** 10.3389/fneur.2022.955378

**Published:** 2022-09-27

**Authors:** Xin-Bin Wang, Hao Dong, Yong-Gang Qiu, Cun-Cheng Lou, De-Yun Huang, Jing Zhang, Di-Hong Chen, Han Feng, Xu Fang

**Affiliations:** ^1^Department of Radiology, Xiaoshan Affiliated Hospital of Wenzhou Medical University, The First People's Hospital of Xiaoshan District, Hangzhou, China; ^2^Department of Cardiology, Xiaoshan Affiliated Hospital of Wenzhou Medical University, The First People's Hospital of Xiaoshan District, Hangzhou, China; ^3^Department of Radiology, Changhai Hospital, Shanghai, China

**Keywords:** cerebral microbleeds, hypertension, computed tomography, nomogram, clinical

## Abstract

**Background:**

Cerebral microbleeds (CMBs) are common in the hypertensive population and can only be detected with magnetic resonance imaging (MRI). The anticoagulation and thrombolytic regimens for patients with >5 CMBs are different from those for patients with ≤ 5 CMBs. However, MRI is not suitable for evaluating CMBs in patients with MRI contraindications or acute ischemic stroke urgently requiring thrombolysis. We aimed to develop and validate a nomogram combining clinical and brain computed tomography (CT) characteristics for predicting >5 CMBs in a hypertensive population.

**Materials and methods:**

In total, 160 hypertensive patients from 2016 to 2020 who were confirmed by MRI to have >5 (77 patients) and ≤ 5 CMBs (83) were retrospectively analyzed as the training cohort. Sixty-four hypertensive patients from January 2021 to February 2022 were included in the validation cohort. Multivariate logistic regression was used to evaluate >5 CMBs. A combined nomogram was constructed based on the results, while clinical and CT models were established according to the corresponding characteristics. Receiver operating characteristic (ROC) and calibration curves and decision curve analysis (DCA) were used to verify the models.

**Results:**

In the multivariable analysis, the duration of hypertension, level of homocysteine, the number of lacunar infarcts (LIs), and leukoaraiosis (LA) score were included as factors associated with >5 CMBs. The clinical model consisted of the duration of hypertension and level of homocysteine, while the CT model consisted of the number of LIs and LA. The combined model consisted of the duration of hypertension, level of homocysteine, LI, and LA. The combined model achieved an area under the curve (AUC) of 0.915 (95% confidence interval [CI]: 0.860–0.953) with the training cohort and 0.887 (95% CI: 0.783–0.953) with the validation cohort, which were higher than those of the clinical model [training cohort: AUC, 0.797 (95% CI: 0.726, 0.857); validation cohort: AUC, 0.812 (95% CI: 0.695, 0.899)] and CT model [training cohort: AUC, 0.884 (95% CI: 0.824, 0.929); validation cohort: AUC, 0.868 (95% CI: 0.760, 0.940)]. DCA showed that the clinical value of the combined model was superior to that of the clinical model and CT model.

**Conclusion:**

A combined model based on clinical and CT characteristics showed good diagnostic performance for predicting >5 CMBs in hypertensive patients.

## Introduction

Cerebral microbleeds (CMBs) are subclinical brain lesions primarily characterized by minor hemorrhage in microvessels (1). CMBs often occur in patients with hypertension or cerebral amyloid angiopathy (CAA) (2). Lee et al. (3) reported that the prevalence of CMBs in a population with hypertension was ~64.7%, mainly located in the thalamus, basal ganglia, cerebellum, and brainstem. CMBs are also associated with intracranial hemorrhages (ICHs), which might increase the risk of hemorrhage complications from anticoagulation therapy or thrombolysis (4, 5). According to previous relevant reviews, patients with ischemic stroke, transient ischemic attack (TIA), or atrial fibrillation can be routinely administered if the number of CMBs is ≤ 5, while for those with >5 CMBs, antiplatelet drugs should be avoided and new oral anticoagulants are recommended (6–8). Furthermore, for acute ischemic stroke patients with >5 CMBs, thrombolysis should be used with caution due to the high risk of ICH (7–9). Thus, it is necessary to determine whether the patient has less than or equal to or more than 5 CMBs in a hypertensive population who are prone to cerebral infarction, TIA, and atrial fibrillation (10–12).

Cerebral microbleeds can be diagnosed with paramagnetic-sensitive MR sequences, such as T2^*^-weighted imaging gradient-recalled echo (GRE-T2^*^WI) or susceptibility-weighted imaging (SWI) sequences (1). However, a part of the population has contraindications to magnetic resonance imaging (MRI), such as an implanted pacemaker or claustrophobia, and MRI is not recommended prior to thrombolytic therapy in patients with acute ischemic stroke because of a delayed thrombolytic time (13). Compared with MRI, brain computed tomography (CT) is more inexpensive and more quickly acquires images and thus can be used for patients with MRI contraindications and those with acute ischemic stroke urgently requiring thrombolysis to exclude the presence of ICH (13). However, to the best of our knowledge, relevant reports about the prediction of the number of CMBs with CT characteristics have not been found.

In this study, we aimed to develop and validate a nomogram combining clinical and CT characteristics to predict whether individuals in a hypertensive population have >5 CMBs; this nomogram can provide clinical value for the formulation of thrombolytic or anticoagulation regimens.

## Materials and methods

### Patients

**Figure 1:**
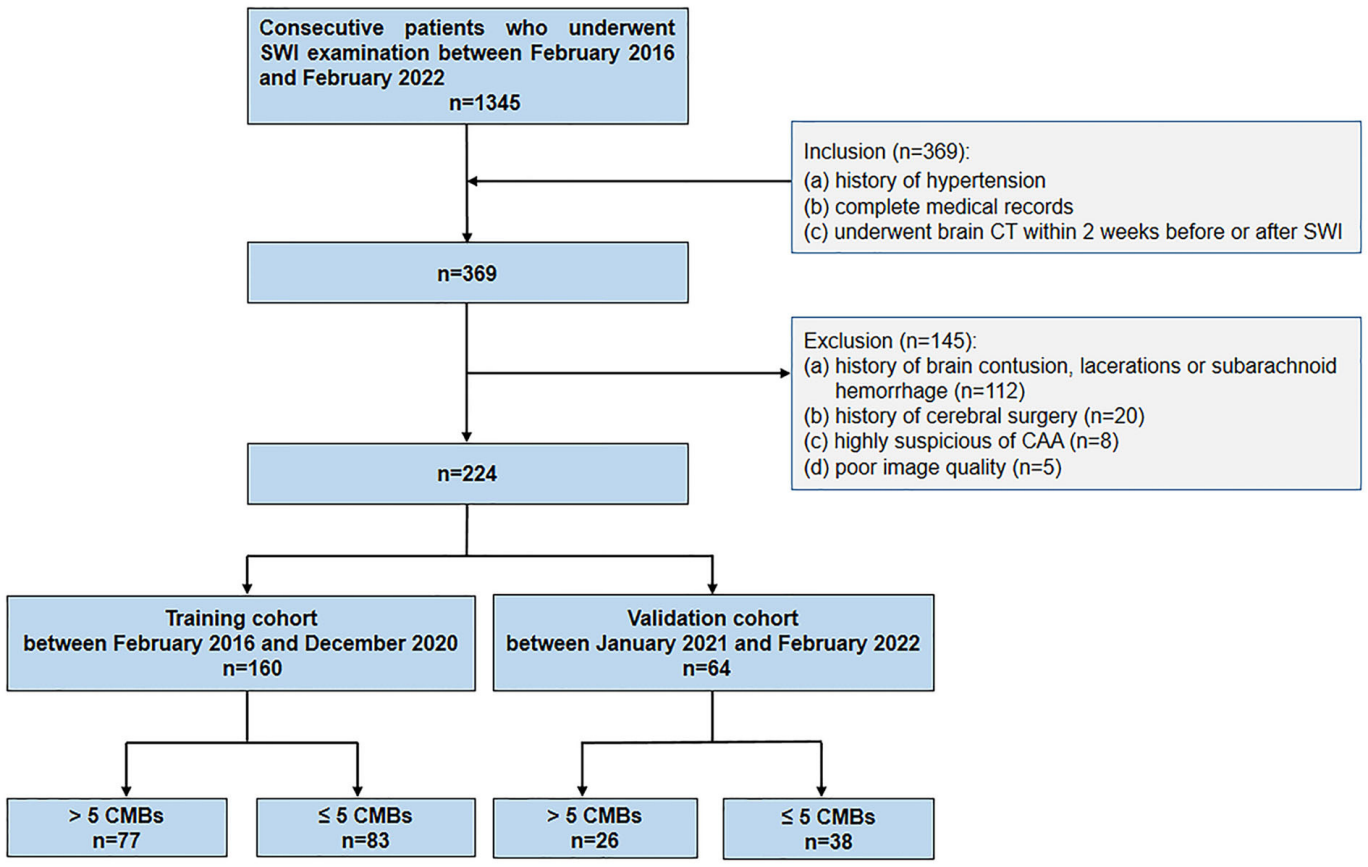
Patient flow diagram.

The Research Ethics Committee of one medical center reviewed and approved this retrospective study. The need for informed patient consent was waived. A total of 224 patients were finally included in this study by searching the medical records from February 2016 to February 2022 in one medical center according to the following inclusion criteria: (1) SWI examination; (2) brain CT performed within 2 weeks of the SWI examination; (3) a history of hypertension; and (4) complete medical data records. The exclusion criteria were as follows: (1) a history of brain parenchymal contusion, lacerations, or subarachnoid hemorrhage (*n* = 112); (2) a history of cerebral surgery (*n* = 20); (3) a high suspicion of CAA according to the Boston criteria (*n* = 8) (14–16); (4) poor CT or SWI image quality (*n* = 5); and (5) Alzheimer's disease, cerebral autosomal dominant arteriopathy with subcortical infarcts and leukoencephalopathy, or multiple sclerosis (*n* = 0). A total of 160 patients whose medical records were obtained from February 2016 to December 2020 constituted the training cohort, and the remaining 64 patients, whose medical records were obtained from January 2021 to February 2022, were included in the validation cohort (Figure 1).

### Collection of clinical and laboratory data

The clinical and laboratory data were collected by screening the institutional medical reports. The clinical data included age, sex, duration of hypertension, grade of hypertension, history of diabetes, coronary heart disease, cerebral infarction, anticoagulant drug use, smoking, and drinking. The laboratory data included high-sensitivity C-reactive protein (HS-CRP; normal range: <8.00 mg/L), triglycerides (normal range: <1.70 mmol/L), total cholesterol (normal range: 3.00–5.70 mmol/L), high-density lipoprotein (normal range: 1.10–1.74 mmol/L), low-density lipoprotein (normal range: 0.00–3.12 mmol/L), platelets (normal range: 125–350 10^9^/L), and homocysteine (normal range: 5.0–15.0 μmol/L).

### Image acquisition

All patients underwent MR examinations using an MR machine (Siemens Aera 1.5 T, GE Signa Voyager 1.5T). SWI was obtained with the following parameters: repetition time (TR): 40 ms, time to echo (TE): 25 ms, slice thickness: 1.8 mm, slice interval: 0 mm, flip angle: 30°, matrix: 320 × 320, and field of view: 230 × 230 mm. SWI images were obtained by transferring the original data into the MRI workstation for postprocessing. Brain CT examinations were performed with a multidetector CT device (Philips Brilliance 64 row; United Imaging 16 row). The scanning parameters for CT were 120 kV tube voltage, 250 mA tube current, field of view: 250 × 250 mm, matrix: 512 × 512, slice thickness 5 mm, and 5-mm slice interval.

### Image analysis

**Figure 2:**
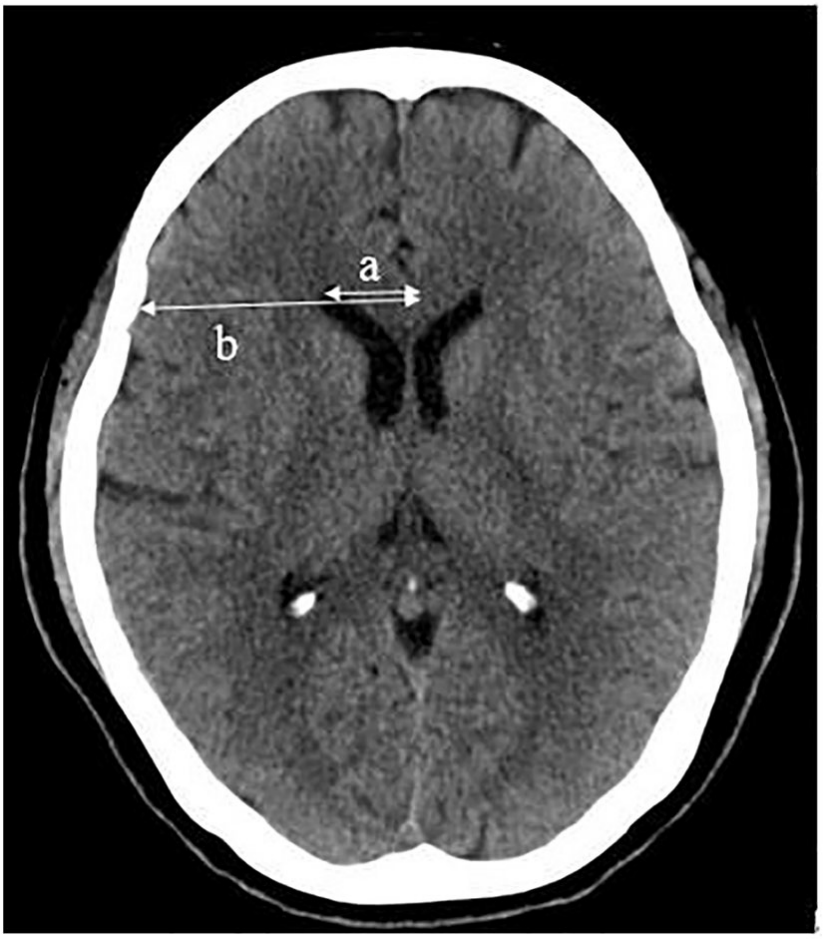
Frontal ratio (%) = a/b × 100, (a: distance from the frontal angle of the lateral ventricle to the cerebrum falx; b: width of the corresponding horizontal frontal lobe).

Susceptibility-weighted imagings were evaluated by two radiologists (with 8 and 12 years of experience in neuroradiology) with the help of artificial intelligence (AI) software (BioMind, Beijing, China), who were unaware of the CT results. CT images were evaluated independently by two board-certified neuroradiologists (with 30 and 32 years of experience in neuroradiology) who were blinded to the SWI results. CMBs were defined as circular and uniform signal loss areas with a diameter of 2–5 mm and a clear edge on SWI images (17). Lacunar infarcts (LIs) were appeared as low-density areas on CT images with a diameter of 3–15 mm in the deep perforating artery territory (18). The degree of leukoaraiosis (LA) on CT images was assessed by the Blennow score, calculated as the average of its extension and intensity (19). The grades of extension of the LA were as follows: no reduction in white matter attenuation = 0; reduced attenuation of white matter located in the occipital and frontal horn edges of the lateral ventricles = 1; reduced attenuation of white matter located around the occipital and frontal horns of the lateral ventricles, with some extending to the semioval center = 2; and reduced attenuation of white matter located around the whole lateral ventricles and merging in the semioval center = 3. The grades of the intensity of LA were no = 0, slight = 1, moderate = 2, and markedly reduced attenuation of white matter = 3 (19). The brain atrophy score was assessed by measuring the frontal ratio (ratio of the distance from the frontal angle of the lateral ventricle to the cerebrum falx to the width of the corresponding horizontal frontal lobe) (Figure 2) (20).

### Statistical analysis

The chi-squared test or Fisher's exact test was used for comparing categorical variables when applicable. The independent-sample *t*-test and the Mann–Whitney *U* test were used for comparing continuous variables when applicable. Interobserver variability was obtained by using an intraclass correlation coefficient (ICC) model, and differences in data were agreed upon negotiation. Multivariate analysis was conducted for statistically significant variables from the univariate analysis and demonstrated the absence of multicollinearity. A combined nomogram was constructed and expressed based on the results, while a clinical model and a CT model were established based on the clinical and CT characteristics, respectively. The receiver operating characteristic (ROC) and calibration curves and decision curve analysis (DCA) were used to verify the model internally and externally. Comparisons between the ROC curves of different models were performed using the Delong nonparametric method. The statistical analyses were performed by using software packages, including SPSS 23.0 (IBM, Armonk, NY, USA), R (v.4.1.2, Vienna, Austria), and MedCalc v. 19.0 (MedCalc Software Bvba). *p* < 0.05 indicated statistical significance.

## Results

### Interobserver agreement

The interobserver ICCs for number of LIs, LA, brain atrophy, and >5 CMBs were 0.873 (95% confidence interval [CI]: 0.838, 0.902), 0.927 (95% CI: 0.905, 0.943), 0.865 (95% CI: 0.828, 0.895), and 0.955 (95% CI: 0.942, 0.965), respectively. All ICCs indicated good agreement.

### Patient characteristics

**Figure 3:**
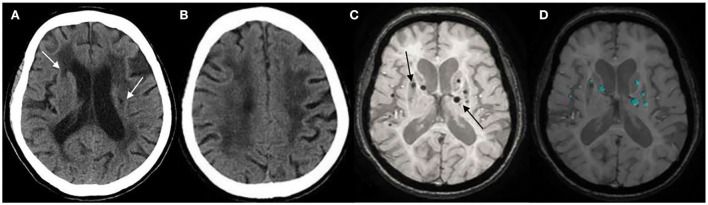
**(A)** Lacunar infarcts (LIs) are seen in the bilateral basal ganglia on computed tomography (CT) (arrow). **(B)** Extensive hypodensity in the bilateral corona radiate, and the Blennow score of the LA was 3. **(C)** susceptibility-weighted imaging (SWI) shows multiple cerebral microbleeds (CMBs; >5) in a 73-years-old female with hypertension for 20 years (arrow). **(D)** CMBs diagnosed by BioMind AI software on SWI (green marker).

Differences in clinical and CT characteristics between patients with >5 CMBs and ≤ 5 CMBs are presented in Table 1. The grade of hypertension, duration of hypertension, homocysteine level, number of LIs, and Blennow score of LA were significantly different between the two groups in the training cohort (*p* < 0.05; Figures 3A–D). Age, duration of hypertension, history of cerebral infarction, history of anticoagulant drugs, number of LIs, and Blennow score of LA showed statistically significant differences between the two groups in the validation cohort (*p* < 0.05). However, there were no significant differences in other characteristics between the two groups.

**Table 1:** Comparison characteristics between ≤ 5 cerebral microbleeds (CMBs) and >5 CMBs group.

**Characteristic**	**Training cohort**			**Validation cohort**		
	** ≤ 5 CMBs** ** (*n* = 83)**	**>5 CMBs** ** (*n* = 77)**	***P*-value**	** ≤ 5 CMBs** ** (*n* = 38)**	**>5 CMBs** ** (*n* = 26)**	***P*-value**
Gender [*n* (%)]			0.194			0.935
Male	40 (48.2)	45 (58.4)		23 (60.5)	16 (61.5)	
Female	43 (51.8)	32 (41.6)		15 (39.5)	10 (38.5)	
Age (year)	64.99 ± 13.20	66.71 ± 12.30	0.392	60.50 ± 10.63	69.96 ± 12.40	0.002
Grade of hypertension [*n* (%)]			0.007			0.115
Grade 1	27 (32.5)	9 (11.7)		12 (31.6)	3 (11.5)	
Grade 2	26 (31.3)	29 (37.6)		8 (21.1)	10 (38.5)	
Grade 3	30 (36.1)	39 (50.6)		18 (47.4)	13 (50.0)	
Duration of hypertension (year)	4.0 (2.0, 8.5)	10.0 (6.0, 16.0)	<0.001	3.0 (2.0, 6.0)	11.0 (4.5, 15.0)	<0.001
Diabetes [*n* (%)]	16 (19.3)	15 (19.5)	0.974	6 (15.8)	3 (11.5)	0.908
Coronary heart disease [*n* (%)]	14 (16.8)	12 (15.6)	0.826	0 (0)	2 (7.7)	0.314
Cerebral infarction [*n* (%)]	27 (32.5)	35 (45.5)	0.095	6 (15.8)	10 (38.5)	0.039
Anticoagulant drugs [*n* (%)]	12 (14.5)	17 (22.1)	0.214	3 (7.9)	8 (30.7)	0.041
Smoking [*n* (%)]	24 (28.9)	28 (36.4)	0.315	16 (42.1)	9 (34.6)	0.546
Drinking [*n* (%)]	19 (22.9)	26 (33.8)	0.126	14 (36.8)	9 (34.6)	0.855
HS-CRP (mg/L)	4.21 (1.53, 9.79)	4.10 (0.77, 8.61)	0.280	6.94 (1.45, 12.10)	3.41 (2.25, 7.55)	0.400
Triglycerides (mmol/L)	1.25 (0.95, 1.69)	1.45 (1.06, 2.26)	0.113	1.23 (0.92, 2.12)	1.55 (0.97, 1.72)	0.897
Total cholesterol (mmol/L)	4.34 ± 1.17	4.19 ± 1.13	0.401	4.30 ± 0.89	4.88 ± 1.59	0.102
High density lipoprotein (mmol/L)	1.28 ± 0.27	1.20 ± 0.25	0.108	1.18 ± 0.25	1.26 ± 0.25	0.209
Low density lipoprotein (mmol/L)	2.53 ± 0.82	2.32 ± 0.87	0.120	2.44 ± 0.76	2.61 ± 0.97	0.425
Platelets (10^9^/L)	213.78 ± 66.52	207.86 ± 63.38	0.563	220.58 ± 75.33	222.54 ± 75.61	0.919
Homocysteine (umol/L)	11.74 ± 3.79	16.59 ± 9.25	<0.001	11.88 ± 9.86	10.91 ± 3.78	0.581
Number of LIs (*n*)	1.0 (0, 3.0)	4.0 (3.0, 6.0)	<0.001	1.5 (0, 3.0)	1.5 (0, 3.5)	0.002
Blennow score of LA [*n* (%)]			<0.001			<0.001
Score 0	43 (51.8)	5 (6.5)		26 (68.4)	4 (15.4)	
Score 1	11 (13.3)	7 (9.1)		4 (10.5)	3 (11.5)	
Score 1.5	3 (3.6)	0 (0)		1 (2.6)	0 (0)	
Score 2	15 (18.1)	16 (20.8)		4 (10.5)	6 (23.1)	
Score 2.5	2 (2.4)	2 (2.6)		0 (0)	1 (3.8)	
Score 3	9 (10.8)	47 (61.0)		3 (7.9)	12 (46.2)	
Brain atrophy score (Frontal ratio, %)	34.52 ± 3.29	34.92 ± 4.38	0.506	34.58 ± 3.710	33.42 ± 3.59	0.220

CMBs, cerebral microbleeds; HS-CRP, high-sensitivity C-reactive protein; LI, lacunar infarcts; LA, leukoaraiosis.

### Nomogram construction

**Figure 4:**
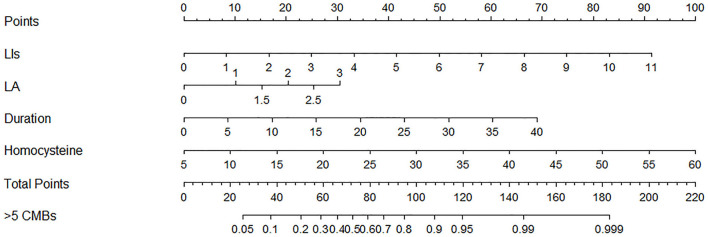
Nomogram combining clinical and computed tomography (CT) characteristics for predicting >5 CMBs in hypertensive patients.

In the univariate analysis, the grade of hypertension, duration of hypertension, homocysteine, number of LIs, and Blennow score of LA were significantly different between the >5 and ≤ 5 CMBs groups in the training cohort. In the multivariate logistic analysis, the duration of hypertension (odds ratio [OR]: 1.114, 95% CI: 1.033–1.200, *p* = 0.005), homocysteine level (OR: 1.120, 95% CI: 1.007–1.246, *p* = 0.037), number of LIs (OR: 1.680, 95% CI: 1.314–2.148, *p* < 0.001), and Blennow score of LA (OR: 1.886, 95% CI: 1.235–2.878, *p* = 0.003) were identified as independent factors predicting >5 CMBs (Table 2). The variance inflation factors (VIFs) of these independent risk factors were all <3, indicating a lack of multicollinearity among them. A clinical model was established by incorporating the duration of hypertension and homocysteine, while a CT model was established by incorporating the number of LIs and LA Blennow score. A combined model was developed by incorporating the four independent factors. Then, a nomogram of the combined model was constructed. First, a weighted score was awarded for each factor. Then, a total score for each hypertension patient was calculated, and the probability of >5 CMBs was analyzed by using the nomogram (Figure 4).

**Table 2:** Univariate and multivariate logistic regression analyses of CT and clinical characteristics.

**Variable**	**Univariate analysis**			**Multivariate analysis**		
	**OR**	**95%CI**	***P***	**OR**	**95%CI**	***P***	**VIF**
Gender	1.512	0.809–2.825	0.194				
Age	1.011	0.986–1.036	0.392				
Grade of hypertension	1.770	1.171–2.675	0.007	1.628	0.878–3.016	0.122	1.066
Duration of hypertension	1.153	1.085–1.225	<0.001	1.114	1.033–1.200	0.005	1.184
Diabetes	1.013	0.462–2.220	0.974				
Coronary heart disease	0.910	0.392–2.112	0.826				
Cerebral infarction	1.728	0.910–3.284	0.095				
Anticoagulant drugs	1.676	0.742–3.787	0.214				
Smoking	1.405	0.723–2.729	0.315				
Drinking	1.717	0.856–3.446	0.126				
HS-CRP	1.006	0.989–1.024	0.471				
Triglycerides	1.043	0.834–1.306	0.710				
Total cholesterol	0.888	0.672–1.172	0.401				
High density lipoprotein	0.367	0.108–1.247	0.108				
Low density lipoprotein	0.733	0.496–1.084	0.120				
Platelets (10^9^/L)	0.999	0.994–1.003	0.563				
Homocysteine	1.176	1.086–1.274	<0.001	1.120	1.007–1.246	0.037	1.126
Number of Lis	1.898	1.550–2.325	<0.001	1.680	1.314–2.148	<0.001	1.450
Blennow score of LA	3.311	2.316–4.733	<0.001	1.886	1.235–2.878	0.003	1.596
Brain atrophy score	15.711	0.005–53027.447	0.506				

CI, confidence interval; OR, odds ratio; VIF, variance inflation factor; LI, lacunar infarcts; LA, leukoaraiosis. Significant parameters with *p* < 0.05 in the univariate analysis were included in the multivariate logistic regression analysis.

### Nomogram performance evaluation

**Figure 5:**
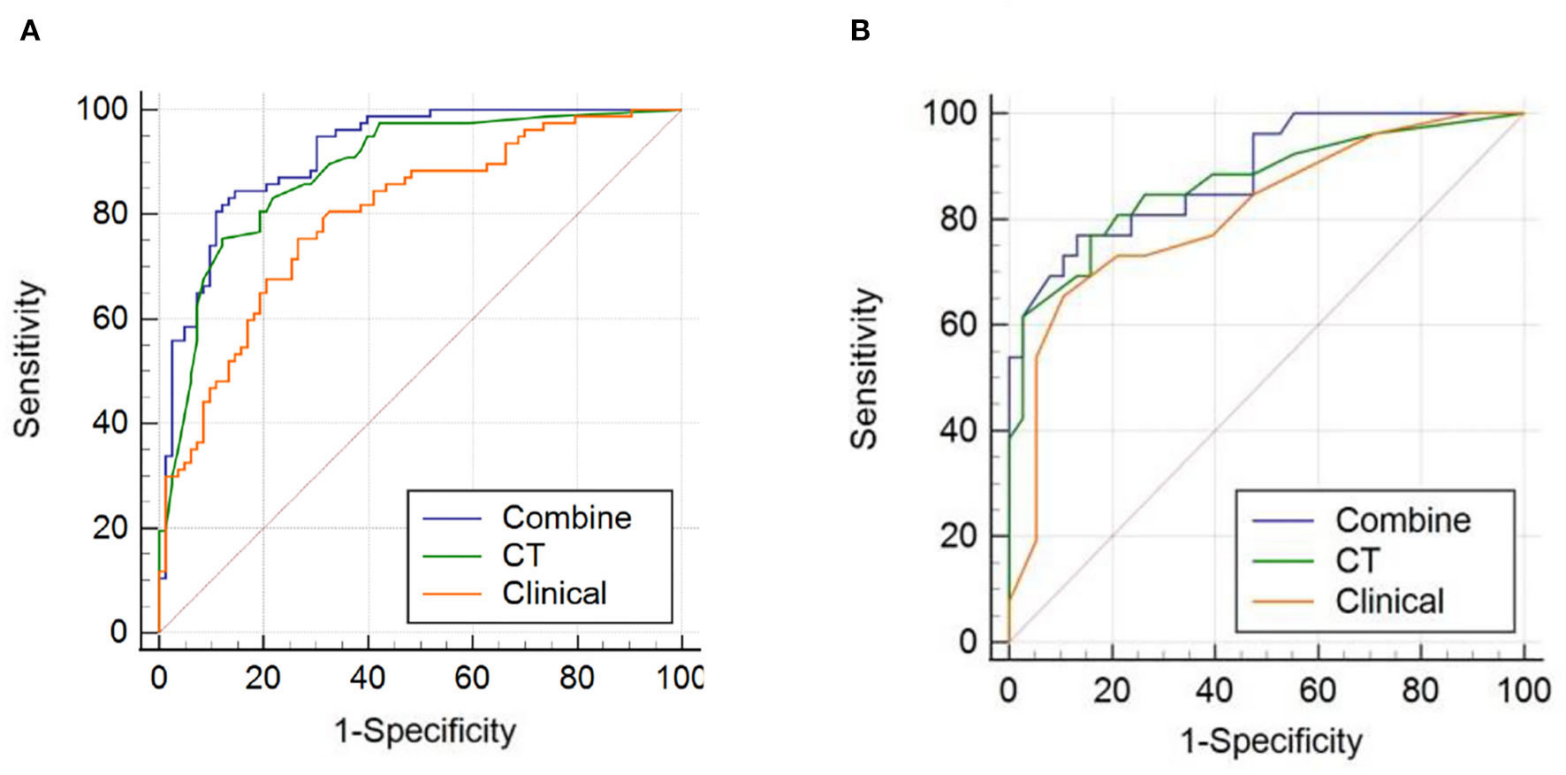
Receiver operating characteristic (ROC) curves of the combined model, computed tomography (CT) model, and clinical model for **(A)** the training cohort and **(B)** the validation cohort.

All ROC curves are shown in Figure 5. For the training cohort, the combined model showed the highest discrimination between the >5 and ≤ 5 CMBs groups, with an area under the curve (AUC) of 0.915 (95% CI: 0.860, 0.953), higher than that of the clinical model (AUC, 0.797 [95% CI: 0.726, 0.857]) and CT model (AUC, 0.884 [95% CI: 0.824, 0.929]). The Delong test showed that the three models were significantly different in the training cohort (combined model vs. clinical model, *p* < 0.001; combined model vs. CT model, *p* = 0.024; and clinical model vs. CT model, *p* = 0.032). In the validation cohort, the combined model yielded the greatest AUC (0.887; 95% CI: 0.783, 0.953), which confirmed that the model achieved better predictive efficacy than the clinical model (AUC, 0.812 [95% CI: 0.695, 0.899]) and CT model (AUC, 0.868 [95% CI: 0.760, 0.940]). The Delong test showed that there were no significant differences among the three models in the validation cohort (combined model vs. clinical model, *p* = 0.169; combined model vs. CT model, *p* = 0.389; and clinical model vs. CT model, *p* = 0.429). Details of the performance of the three models are shown in Table 3.

**Table 3:** Predictive performance of clinical model, CT model, and combined model.

**Model**	**Training cohort**			**Validation cohort**		
	**AUC (95%CI)**	**Specificity**	**Sensitivity**	**AUC (95%CI)**	**Specificity**	**Sensitivity**
Clinical model	0.797 (0.726–0.857)	0.735	0.753	0.812 (0.695–0.899)	0.895	0.654
CT model	0.884 (0.824–0.929)	0.880	0.753	0.868 (0.760–0.940)	0.842	0.769
Combined model	0.915 (0.860–0.953)	0.855	0.844	0.887 (0.783–0.953)	0.868	0.769

CI, confidence interval; AUC, area under the curve.

**Figure 6:**
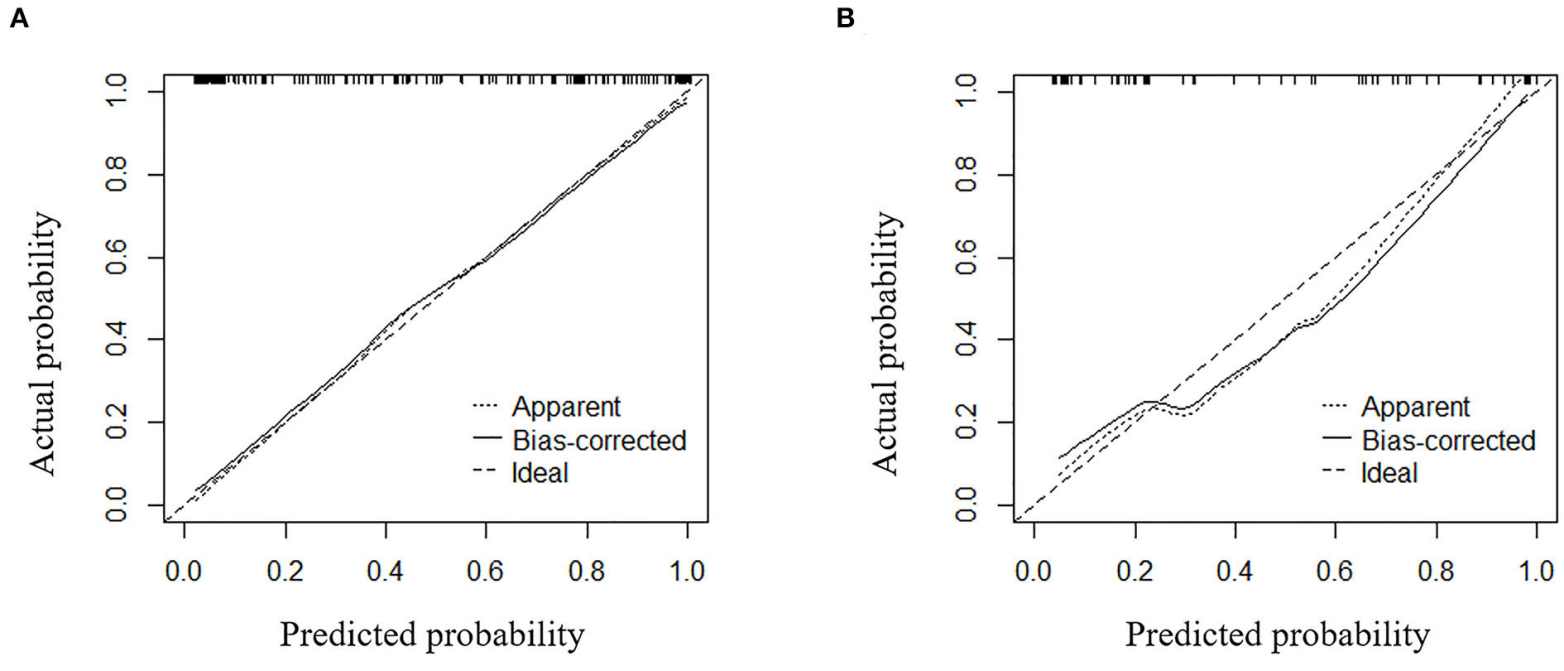
Calibration curves for the combined model used for predicting >5 CMBs for **(A)** the training cohort and **(B)** the validation cohort.

The calibration curve of the nomogram demonstrated good agreement between the predicted and observed proportions of patients with >5 CMBs in the training cohort (Figure 6A). The Hosmer-Lemeshow test for the training cohort yielded a *p*-value of 0.401, suggesting that the nomogram had a perfect fit. The calibration of the nomogram was further confirmed with the validation cohort (Figure 6B), for which the Hosmer-Lemeshow test yielded a *p*-value of 0.199, suggesting no departure from the good fit of the nomogram.

**Figure 7:**
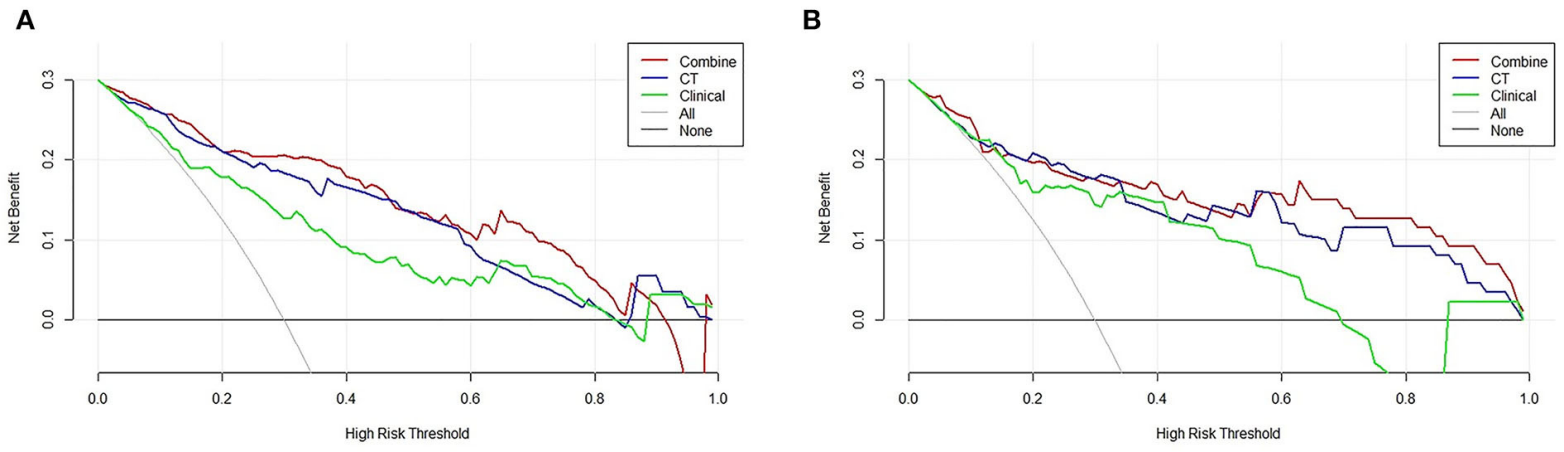
Decision curve analysis (DCA) of the combined model, clinical model, and computed tomography (CT) model. Evaluation in **(A)** the training cohort and **(B)** the validation cohort. *y*-axis: net benefit; *x*-axis: threshold probability. The combined model (red line) obtained the highest net benefit compared with the CT model (blue line), the clinical model (green line), the intervention-all strategy (gray line), and the intervention-none strategy (horizontal black line).

**Figure 8:**
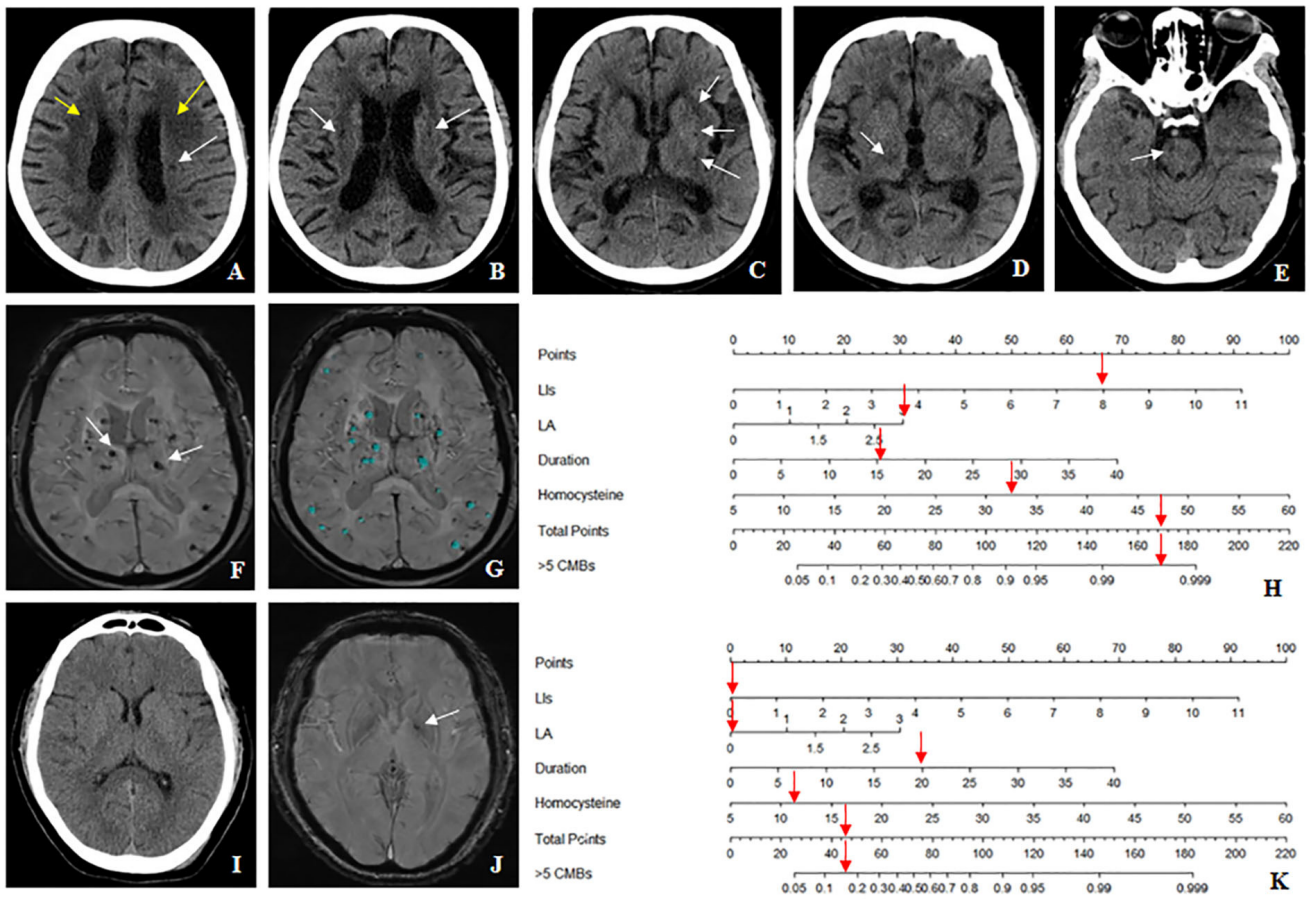
The nomogram accurately differentiates >5 cerebral microbleeds (CMBs) with ≤ 5 CMBs in the hypertensive population. **(A–H)** Case 1: a 64-years-old female with hypertension for 15 years, whose homocysteine level was 32.9 μmol/L. **(A)** The Blennow score of the LA was 3 (yellow arrow). **(A–E)** The number of LIs defined by CT was 8 located in the left basal ganglia, right thalamus, bilateral semiovale center, and brainstem region (white arrow). **(F,G)** susceptibility-weighted imaging (SWI) shows multiple CMBs (>5) (white arrow), which were diagnosed by BioMind AI software (green marker). **(H)** According to the combined model, the prediction probability of >5 CMBs was more than 99% (red arrow). **(I–K)** Case2: a 54-years-old male with hypertension for 20 years, whose homocysteine level was 11.1 μmol/L. **(I)** Both the Blennow score of the LA and the number of LIs were 0. **(J)** Only one CMB was seen in the left basal ganglia region (white arrow), which met the diagnostic criteria of ≤ 5 CMBs. **(K)** According to the combined model, the prediction probability of >5 CMBs was between 10 and 20% (red arrow).

Decision curve analysis showed that the curve of the combined model (red) was above that of the CT model (blue) and the clinical model (green) both in the training cohort and validation cohort over a large range of threshold probabilities, suggesting that the combined model had a higher overall net benefit in identifying patients with ≤ 5 and <5 CMBs than both the CT model and the clinical model (Figures 7A,B). The nomogram drawn according to the predictive model was used in practical cases, and its predictive efficiency had been confirmed (Figure 8).

## Discussion

This study retrospectively analyzed the clinical and CT characteristics of hypertensive patients to predict the probability that they had >5 CMBs to assist physicians in clinical decision-making. Four predictors were screened to build the model in this study, namely, duration of hypertension, homocysteine level, number of LIs, and LA score. The combined model had good predictive performance in both the training cohort and the validation cohort, and the areas under the ROC curve were 0.915 and 0.887, respectively, which were higher than those of the clinical model (training cohort: 0.797; validation cohort: 0.812) and CT model (training cohort: 0.884; validation cohort: 0.868). The Hosmer-Lemeshow test showed that the combined model had a good fit in both the training cohort and the validation cohort. DCA confirmed that the combined model was beneficial for making clinical decisions and provided superior benefit to the clinical model and CT model.

In a previous study, Henskens et al. (21) found a correlation between CMBs and the duration of hypertension, which is consistent with our study. The occurrence of CMBs was related to the mechanism of hypertension aggravating the intimal injury of small intracranial vessels (2). Therefore, a longer history of hypertension led to more CMBs. In this study, the multivariate logistic analysis showed that homocysteine was an independent factor predicting >5 CMBs. Homocysteine is highly related to cerebrovascular and cardiovascular diseases (22–25); a high homocysteine level damages the functional and structural integrity of endothelial cells and promotes the development of atherosclerosis, causing an increased risk of bleeding from small blood vessels (26). This might explain one of the results of our study. However, previous studies found that the grade of hypertension was a risk factor for the occurrence of CMBs, but this factor was excluded in the multivariate analysis in our study (27, 28). We speculate that this might be related to the different ways in which the number of CMBs was grouped, leading to differences in some factors.

We developed a combined model including two clinical characteristics (duration of hypertension and homocysteine level) and two CT characteristics (the number of LIs and the LA score) for predicting >5 CMBs in the hypertensive population. To the best of our knowledge, relevant reports about similar models have not been found, indicating the uniqueness of our findings. In our study, the AUCs of the combined model in the training cohort and the validation cohort were 0.915 and 0.887, respectively, indicating that this model has high diagnostic performance. Furthermore, the AUC of the combined model was higher than that of the CT model and the clinical model for both the training and validation cohort, which showed that the combined model had the best prediction effect. However, the Delong test showed that the differences in the ROC curves for the validation cohort were not significantly different among the three models. We speculated that the reason might be related to the small amount of data and the low sensitivity of the combined model in the validation cohort. In addition, when compared with MRI, CT is widely used in primary hospitals because it is less expensive, has a faster image acquisition speed, and could be applied to patients with MRI contraindications; it is also a necessary examination for patients with acute cerebral infarction for excluding ICH. In our study, the number of LIs and the LA score, both evaluated by CT, were associated with CMBs, similar to the conclusions of previous multisequence MRI studies, which all describe a mechanism of endothelial injury of intracranial small vessels caused by long-term hypertension (29–33). Additionally, clinical data could be obtained by obtaining a patient's history or drawing blood. Therefore, the CT and clinical data used to establish the combined model were easy to obtain at a lower cost and provided better results.

Our study had several limitations. First, selection bias might be present because of the retrospective design of our study. We hope to conduct prospective studies in the future. Second, this was a single-center study without an external validation cohort. Thus, we should focus on multicenter studies to validate the results with external datasets in the future. Third, only recent antihypertensive control was recorded in the medical system, and there was a lack of follow-up records for long-term antihypertensive control. We hope to collaborate with cardiologists and further search the literature to explore indicators of long-term antihypertensive control and apply them to our study. Fourth, our inclusion criteria did not allow populations requiring anticoagulation or thrombolysis because of the small sample size. We would like to expand this study by only including the hypertensive population with ischemic stroke, TIA, or atrial fibrillation in order to better approximate clinical settings in which the sample size is sufficient. Fifth, hemoglobin A1C (HbgaA1C) level may be a more accurate factor as a measure of diabetes control rather than the presence/absence of diabetes. However, it was excluded because the data on HbgaA1C level of some patients are missing. We will include the HbgaA1C level when the sample size is large enough in the future study.

In conclusion, we established a combined model for predicting >5 CMBs in a hypertensive population by incorporating four critical characteristics. This model demonstrated good discriminability and thus could provide substantial assistance for clinical practice, especially for the formulation of antithrombotic regimens for patients with contraindications to MRI or those in primary hospitals not equipped with MRI and for the formulation of thrombolytic regimens for patients with acute ischemic stroke who do not have enough time to undergo MRI.

## Data availability statement

The original contributions presented in the study are included in the article/supplementary material, further inquiries can be directed to the corresponding author.

## Ethics statement

The studies involving human participants were reviewed and approved by the Institutional Review Boards of Xiaoshan District First People's Hospital. Written informed consent for participation was not required for this study in accordance with the national legislation and the institutional requirements.

## Author contributions

X-BW contributed to data analysis and manuscript editing. HD, Y-GQ, C-CL, D-HC, and HF helped in images analysis. D-YH and JZ helped in collecting clinical data. XF helped critically revise the manuscript for important intellectual content. All authors contributed to the article and approved the submitted version.

## Funding

This research was supported by the Major Science and Technology Project of Xiaoshan District (Grant No. 2019214), the Medical and Health Science and Technology Project of Hangzhou (Grant No. B20210068), and 234 Platform Discipline Consolidation Foundation Project (2020YPT001).

## Conflict of interest

The authors declare that the research was conducted in the absence of any commercial or financial relationships that could be construed as a potential conflict of interest.

## Publisher's note

All claims expressed in this article are solely those of the authors and do not necessarily represent those of their affiliated organizations, or those of the publisher, the editors and the reviewers. Any product that may be evaluated in this article, or claim that may be made by its manufacturer, is not guaranteed or endorsed by the publisher.
